# Impact of Xenobiotic Detoxification Gene Polymorphisms on Steady-State Plasma Concentrations of Apixaban and the Development of Hemorrhagic Complications in Older Patients with Non-Valvular Atrial Fibrillation

**DOI:** 10.3390/genes16101179

**Published:** 2025-10-10

**Authors:** Andrey P. Kondrakhin, Sherzod P. Abdullaev, Ivan V. Sychev, Pavel O. Bochkov, Svetlana N. Tuchkova, Karin B. Mirzaev, Maksim L. Maksimov, Dmitry A. Sychev

**Affiliations:** 1 Moscow Healthcare Department, War Veterans Hospital No. 2, Moscow 109472, Russia; a.kondrachin@yandex.ru; 2 Federal State Budgetary Research Institution «Russian Research Center of Surgery Named After Academician B.V. Petrovsky», Moscow 119991, Russia; sychev_iv@bk.ru (I.V.S.); svetlana.tuch1998@gmail.com (S.N.T.); karin05doc@yandex.ru (K.B.M.); dimasychev@mail.ru (D.A.S.); 3 Federal State Budgetary Educational Institution, Ministry of Healthcare of the Russian Federation, Further Professional Education “Russian Medical Academy of Continuous Professional Education”, Moscow 125993, Russia; bok-of@yandex.ru (P.O.B.); maksim_maksimov@mail.ru (M.L.M.)

**Keywords:** atrial fibrillation, apixaban, gene, pharmacogenetics, direct oral anticoagulants, ABCB1 polymorphism, rs1045642, bleeding risk

## Abstract

Background: Atrial fibrillation (AF) is the most common sustained cardiac arrhythmia and is associated with a fivefold increase in stroke risk. Direct oral anticoagulants (DOACs), including apixaban, are now the preferred therapy for stroke prevention in patients with non-valvular AF (NVAF). However, interindividual variability in drug response and safety remains a major challenge, particularly in elderly patients with comorbidities and polypharmacy. Genetic polymorphisms in drug-metabolizing enzymes and transporters may contribute to variability in apixaban exposure and bleeding risk. This study aimed to evaluate the association of polymorphisms in *ABCB1*, *CYP3A4*, and *CYP3A5* with steady-state plasma concentrations of apixaban (Cssmin) and hemorrhagic complications in elderly patients with NVAF. Methods: This cross-sectional study included 197 patients (mean age 83 ± 8 years; 67% women) with NVAF treated with apixaban (5 mg twice daily). Genotyping of *ABCB1* (rs1045642, rs2032582, rs1128503), CYP3A4*22 (rs35599367), and CYP3A5*3 (rs776746) was performed using allele-specific real-time PCR. Cssmin of apixaban was determined by high-performance liquid chromatography coupled with tandem mass spectrometry. Associations with bleeding events were evaluated. Results: Bleeding events were recorded in 40 patients (20.3%). An association signal was observed for *ABCB1* rs1045642, where carriers of the CC genotype had a higher risk of bleeding compared with alternative alleles (OR = 2.805; 95% CI: 1.326–5.935; *p* = 0.006). After correction for multiple testing, the association remained significant only under the log-additive model (OR = 1.93 per C allele; 95% CI: 1.17–3.20; q = 0.0275; p_adj = 0.044), while recessive and codominant effects did not withstand Bonferroni adjustment. No significant associations were observed for rs2032582, rs1128503, CYP3A4*22, or CYP3A5*3. None of the studied polymorphisms, including rs1045642, significantly affected Cssmin. Concomitant therapy, particularly with antiarrhythmic drugs and statins (rosuvastatin), also increased bleeding risk. Conclusions: The findings highlight the potential contribution of ABCB1 rs1045642 and specific drug–drug interactions to the risk of hemorrhagic complications in elderly NVAF patients receiving apixaban.

## 1. Introduction

Atrial fibrillation (AF) is the most common form of sustained cardiac arrhythmia and continues to exert a progressive and substantial impact on global public health. According to the Global Burden of Disease study, the prevalence of AF is estimated at 59.7 million cases worldwide [1]. The prevalence of AF increases sharply with age: it is estimated to affect 5–10% of individuals aged ≥ 65 years and more than 10% of those over 80 years [2,3]. AF also increases the risk of stroke fivefold in patients regardless of age [4,5]. In addition, patients with AF frequently present with a comorbid status that includes arterial hypertension, coronary artery disease, heart failure, chronic kidney disease, and other conditions.

According to current guidelines, direct oral anticoagulants (DOACs) are recommended as the preferred agents for the prevention of thromboembolic complications in patients with AF, except for those with mechanical heart valves or severe mitral stenosis, in whom warfarin remains the standard anticoagulant [6,7]. Unlike warfarin, which inhibits multiple coagulation factors, DOACs directly inhibit thrombin or factor Xa, the final stage of the coagulation cascade. DOACs are administered in fixed doses, have fewer drug–drug interactions, and exhibit more stable pharmacokinetics, thereby eliminating the need for regular INR monitoring—an important advantage over warfarin. DOACs are metabolized through common phase I and II biotransformation enzymes, including cytochrome P450 isoenzymes and esterases. For instance, dabigatran etexilate, being a prodrug, is activated in the body by the CES1 enzyme, whereas rivaroxaban and apixaban are predominantly metabolized by CYP3A4 and CYP3A5 [8]. Another key player in ADME processes is the P-glycoprotein transporter. Polymorphisms in genes encoding these enzymes and transporters can significantly affect plasma drug concentrations, altering the therapeutic index and predisposing patients to adverse outcomes, including bleeding or thrombosis [9]. For apixaban, population PK/pharmacogenomic analyses and candidate-gene studies have explored associations between transporter and metabolizing enzyme variants and exposure to, or bleeding from, the medication. In Japanese AF cohorts, CYP3A5*3 (rs776746) and *ABCG2* 421A/A have been linked to higher concentration-to-dose ratios, whereas common *ABCB1* variants (1236C>T, 2677G>T/A, 3435C>T) generally showed null or inconsistent PK effects [10,11]. In predominantly European cohorts, Lenoir et al. (2022) and Attelind et al. (2022) did not observe effects of the three ABCB1 SNPs on apixaban AUC or plasma levels [12,13]. Meta-analyses report heterogeneous findings: Xie et al. (2018) and Shi et al. (2023) found modest associations of rs1045642 with PK metrics across DOACs, while clinical bleeding associations remain inconsistent across settings [14,15]. Recent clinical data suggest a possible link between *ABCB1* rs1045642 and bleeding in DOAC-treated patients after adjustment for confounders, though replication is needed and effect sizes are modest; other cohorts report no association [16,17]. Although the impact of CYP gene variants and drug transporter polymorphisms on DOAC exposure and clinical outcomes (bleeding and thrombotic events) has been investigated, the findings remain inconsistent [9,13,18].

Genetic polymorphisms contribute to interindividual variation in therapeutic response, while comorbidities and multiple concomitant medications may further perturb drug pharmacokinetics and narrow the individual therapeutic window [19]. The risk of complications is elevated in patients with chronic kidney disease, diabetes, or those exposed to drugs metabolized by overlapping hepatic or transporter pathways. In geriatric settings, the confluence of multimorbidity and polypharmacy amplifies adverse outcome risk, underlining the importance of tailored therapy and monitoring [20]. 

The aim of this study was therefore to evaluate the impact of genetic polymorphisms in *ABCB1*, *CYP3A4*, and *CYP3A5*—encoding P-glycoprotein and major drug-metabolizing enzymes—on the variability of apixaban plasma concentrations and the risk of hemorrhagic complications in elderly patients with non-valvular atrial fibrillation (NVAF) receiving polypharmacy.

## 2. Materials and Methods

### 2.1. Ethical Approval

The study was approved by the Local Ethics Committee of the War Veterans Hospital No. 2 of the Moscow Healthcare Department (Protocol No. 226, 20 February 2023). This study was conducted in accordance with the legislation of the Russian Federation and international regulatory documents (the Declaration of Helsinki of the World Medical Association, 2013; National Standard of the Russian Federation GOST R 52379-2005).

All patients who participated in the study provided written informed consent. Before obtaining consent, the study protocol, potential risk factors, and other relevant aspects were explained in detail to each patient, who was also given the opportunity to ask questions.

### 2.2. Study Population

Study Design: this was a cross-sectional study that included patients hospitalized at the War Veterans Hospital No. 2 of the Moscow Healthcare Department between April and September 2023

Inclusion Criteria were as follows:

1. Age ≥ 65 years;
2. Non-valvular atrial fibrillation (NVAF)
3. Chronic kidney disease (CKD) stage ≤ 4;
4. Polypharmacy, defined as the concurrent use of more than five medications;
5. Signed written informed consent

Non-inclusion Criteria included the following:

1. Dementia precluding provision of valid informed consent (requiring a legal representative;
2. CKD stage 5;
3. Acute coronary or cerebrovascular pathology at enrollment;
4. History of myocardial infarction, stroke, or cardiac surgery;
5. Clinically significant valvular heart disease (rheumatic or other etiology);
6. Chronic heart failure stage IIB or higher;
7. Alcohol-induced atrial fibrillation;
8. Active gout, thyroid dysfunction, exacerbated gastrointestinal diseases, hepatic impairment, oncological disease, or active inflammatory process of any localization;
9. Hematological disorders.

Exclusion criteria were:

1. Contraindications to apixaban;
2. Refusal to adhere to prescribed pharmacotherapy or withdrawal of consent.

A total of 197 patients with NVAF receiving apixaban (5 mg twice daily with meals) were included in the study. The mean age was 83 ± 8 years; the cohort consisted of 132 women (67%) and 65 men (33%).

For each patient, the individual case report form included the following data: sex, age, weight, height, CKD stage, creatinine clearance calculated using the Cockcroft-Gault formula, indication for DOAC therapy, total duration of DOAC use, results of complete blood count, and complications related to DOAC therapy. In accordance with international criteria, CKD was defined as a reduction in glomerular filtration rate to ≤60 mL/min/1.73 m^2^ for a duration of at least three months.

The severity of bleeding was evaluated based on the definition provided by the International Society on Thrombosis and Hemostasis. In brief, events leading to a reduction in hemoglobin levels by at least 2 g/dL, fatal bleeding, or events occurring in critical body locations were considered major bleeding events. Events that required hospitalization, medical or surgical intervention, or a change in antithrombotic treatment were considered clinically relevant non-major. Any acute bleeding event not meeting the criteria for major or clinically relevant non-major bleeding was classified as a minor bleeding episode.

### 2.3. Genotyping

On the day of study enrollment, 5 mL of blood was collected from each patient into single-use sterile vacuum tubes containing EDTA for subsequent genotyping. Blood sampling was performed simultaneously with routine laboratory tests and did not require additional venipunctures. The samples were frozen at –20 °C, transported to the laboratory, and stored at –70 °C until analysis.

DNA was extracted from venous blood using a column-based method with the QIAamp DNA Blood Mini Kit (Qiagen, Hilden, Germany). DNA concentration and quality were assessed with a Nanodrop ND-1000 spectrophotometer (Thermo Fisher Scientific, Waltham, MA, USA).

In all enrolled patients, allele-specific real-time PCR was used to identify polymorphic variants of *ABCB1* (rs1045642 3435C>T, rs2032582 2677G>T/A, rs1128503 1236C>T), CYP3A4*22 (rs35599367), and CYP3A5*3 (rs776746, 6986A>G). Commercial kits (OOO Syntol, Moscow, Russia) were employed on a CFX96 Touch™ Real-Time PCR Detection System (Bio-Rad, Hercules, CA, USA).

The choice of polymorphisms was primarily based on their functional relevance to apixaban disposition and prior pharmacogenetic evidence. The three common ABCB1 variants (rs1045642, rs2032582, rs1128503) form a haplotype block associated in some studies with altered P-glycoprotein function. CYP3A4*22 (rs35599367) reduces CYP3A4 expression, while CYP3A5*3 (rs776746) abolishes CYP3A5 activity. These variants have been linked, albeit inconsistently, to DOAC pharmacokinetics and bleeding risk. Their allele frequencies are relatively common in European populations, making them suitable for analysis in our Russian cohort.

### 2.4. Apixaban Plasma Determination

For all patients, the minimum steady-state plasma concentration of apixaban (Cssmin) was determined. Plasma samples were collected after at least five days of continuous DOAC therapy. Plasma samples were obtained by centrifuging whole blood at 3000 rpm for 15 min. The separated plasma was aliquoted into Eppendorf tubes and stored at –70 °C until analysis. Quantification of apixaban plasma concentrations was performed using high-performance liquid chromatography coupled with tandem mass spectrometry (HPLC–MS/MS) on an Agilent 1200 chromatograph (equipped with a quaternary pump, degasser, and thermostatted column compartment) (Agilent Technologies Inc., Santa Clara, CA, USA, 2008) with detection by an Agilent Triple Quad LC/MS 6410 mass spectrometer (triple quadrupole).

Sample preparation was carried out by protein precipitation. Frozen plasma samples were thawed at room temperature, and 100 μL of plasma was transferred into Eppendorf tubes. Subsequently, 250 μL of a methanol/0.1% HCl mixture (9:1, *v*/*v*) was added, vortex-mixed, left to stand for 10 min, and vortex-mixed again. The mixtures were centrifuged at 10,000 rpm for 10 min, and the supernatant was transferred to chromatographic vials and placed in the autosampler.

Chromatographic separation was performed on an Agilent Extend-C18 column (100 mm × 2.1 mm, 3.5 μm) at 40 °C. The mobile phase consisted of solvent A (50 mL of 0.1 M ammonium acetate solution and 5 mL of formic acid diluted with deionized water to a final volume of 1 L) and solvent B (50 mL of 0.1 M ammonium acetate solution and 5 mL of formic acid diluted with acetonitrile to 1 L). Isocratic elution was applied at an A–B ratio of 70:30, with a flow rate of 0.3 mL/min. The injection volume was 10 μL, and the total run time was 7 min. Apixaban detection was performed in multiple reaction monitoring (MRM) mode using electrospray ionization (positive mode). The following parameters were applied: nebulizer gas pressure 35 psi, drying gas flow 11 L/min at 350 °C, fragmentor voltage 135 V, and collision energy 25 V.

The developed method satisfies acceptance criteria and can be used for the quantitative determination of apixaban in blood plasma samples in a pharmacokinetic study.

### 2.5. Statistical Analysis

The study data were subjected to statistical processing using both parametric and nonparametric methods. Statistical analyses were performed with the standard software package StatSoft Statistica 10.0 (Dell, Round Rock, TX, USA). Quantitative variables were assessed for normality using the Kolmogorov–Smirnov test (for sample sizes > 50), as well as by evaluating skewness and kurtosis. For quantitative variables with a normal distribution, the data were organized into variational series, and arithmetic means (M) with standard deviations (SD) were calculated. Comparisons of means in normally distributed quantitative datasets were performed using Student’s *t*-test, with obtained values evaluated against critical thresholds. For categorical variables, differences in frequency distributions were assessed using the χ^2^ test, and Fisher’s exact test was applied when the number of observations was small. To quantify the effect of genetic markers on complication occurrence, odds ratios (ORs) with 95% confidence intervals (CIs) were calculated. Statistical significance for all analyses was defined at *p* < 0.05. For genetic analyses, multiple-testing correction was performed using the Benjamini–Hochberg false discovery rate (FDR) procedure. 

## 3. Results

### 3.1. Analysis of Clinical, Demographic, and Laboratory Parameters

The demographic and clinical characteristics of the study population are presented in Table 1.

Anthropometric indicators were within the ranges expected for an elderly cohort: the mean height was 1.64 ± 0.09 m, body weight 75.5 ± 16.9 kg, and body mass index (BMI) 28.0 ± 5.6 kg/m^2^, corresponding to the overweight range.

Renal function indicators demonstrated a reduction in glomerular filtration rate (GFR), consistent with chronic kidney disease (CKD) stage ≥ 2. The mean GFR in the study population was 49.3 ± 21.2 mL/min/1.73 m^2^, confirming that the cohort predominantly consisted of patients with moderate renal impairment. The mean duration of apixaban therapy at the time of enrollment was 1.95 ± 0.40 years, enabling the assessment of both short-term and long-term treatment-related complications.

According to laboratory parameters, patients exhibited moderate age-related changes. The mean hemoglobin level was 118 ± 19 g/L, indicating a high prevalence of mild anemia in the studied cohort. The mean leukocyte count was 6.66 ± 2.38 × 10^9^/L, platelet count 224 ± 75 × 10^9^/L, and erythrocyte count 4.13 ± 0.64 × 10^12^/L, all generally within normal ranges. The mean urea and creatinine levels were 9.57 ± 9.39 mmol/L and 106 ± 34 μmol/L, respectively, reflecting the presence of concomitant CKD.

In 40 patients (20.3%), bleeding events of varying severity were recorded. The majority were clinically minor episodes (epistaxis—31 cases; hematomas and ecchymoses—3 cases), whereas clinically significant events occurred less frequently (uterine bleeding—1 case; gastrointestinal bleeding—3 cases; peptic ulcer exacerbation—1 case; and a bleeding event in the context of surgery—1 case).

A comparative analysis of clinical, demographic, and laboratory parameters between patients with and without bleeding events showed that the groups were comparable in terms of age, sex, anthropometric characteristics, renal function, and duration of apixaban therapy (*p* > 0.05).

In the overall cohort, the only statistically significant difference was observed in platelet counts: patients with bleeding events had significantly lower levels (218.29 ± 69.53 × 10^9^/L vs. 246.30 ± 92.55 × 10^9^/L, *p* = 0.035). This difference was more pronounced among men, where the mean platelet count in the bleeding subgroup was substantially lower compared with men without bleeding events (198.39 ± 57.41 × 10^9^/L vs. 260.71 ± 142.78 × 10^9^/L, *p* = 0.015). No such association was identified among women.

It is noteworthy that women with bleeding events tended to have lower height and body weight compared with women without complications, although these differences did not reach statistical significance. In men, body weight and BMI values were nearly identical between groups. This may suggest that in the female cohort, additional factors (such as lower body weight and smaller volume of distribution) could potentially increase apixaban exposure and elevate the risk of bleeding.

Laboratory parameters did not show statistically significant differences between the groups. However, men with bleeding events exhibited a tendency toward higher creatinine levels, which may reflect a greater burden of concomitant renal pathology, although this difference did not reach statistical significance.

When analyzed by sex, the likelihood of bleeding events was similar in men and women: the odds ratio (OR) was 1.119 (95% CI: 0.539–2.324; *p* = 0.763), indicating no significant association between sex and the frequency of hemorrhagic complications.

As part of the study, the association between CKD stage and the frequency of hemorrhagic complications in patients receiving apixaban therapy was analyzed (Table 2). Overall analysis demonstrated that the frequency of bleeding events did not depend on the severity of renal impairment: the cumulative assessment of the association between CKD stage and bleeding risk was statistically non-significant (χ^2^ = 1.082; *p* = 0.782).

When individual CKD stages were considered, no significant differences were observed: the frequency of bleeding events was comparable across CKD stages 2–4. Although a trend toward a higher frequency of complications was noted in stage 3a, the difference did not reach statistical significance (OR = 1.427; 95% CI: 0.697–2.920).

### 3.2. Genotyping Results

The distribution of genotypes for the investigated polymorphisms was consistent with the Hardy–Weinberg equilibrium (*p* > 0.05) (Table 3).

For the *ABCB1* gene, minor allele frequencies ranged from 42.1% for rs1128503 to 52.3% for rs1045642, which is consistent with population data from European cohorts [21]. The most balanced genotype distribution was observed for rs1045642, where the proportion of heterozygous carriers (CT) accounted for nearly half of the sample (46.9%). For the CYP3A5*3 (rs776746) polymorphism, there was a marked predominance of homozygotes for the minor G allele (85.05%), whereas the proportion of A allele carriers was extremely low (0.52%), reflecting the reduced expression of functional CYP3A5 enzyme typical of European populations, which may potentially decrease the metabolism of CYP3A5 substrates, including apixaban. The CYP3A4*22 (rs35599367) polymorphism was rare in this cohort: The vast majority of patients (94.5%) carried the CC genotype, and only 5.5% were heterozygous (CT). No homozygotes for the T allele were identified, which is also consistent with the reported low prevalence of this variant in European populations.

Analysis of the distribution of bleeding frequency by genotypes of the studied polymorphisms revealed a significant association only for the *ABCB1* rs1045642 (3435C>T) variant (Table 4).

In the unadjusted analysis, patients with the CC genotype of rs1045642 had an association with a higher risk of hemorrhagic complications compared with carriers of alternative alleles (OR = 2.805; 95% CI: 1.326–5.935; *p* = 0.006). However, this association did not remain statistically significant after correction for multiple testing (FDR or Bonferroni).

In addition, an analysis of the association between rs1045642 and bleeding events was performed under different inheritance models (codominant, dominant, recessive, and additive) (Table 5).

In the codominant model, CC carriers had a higher risk compared with T allele carriers (OR = 3.50; 95% CI: 1.30–9.45; *p* = 0.027). In the recessive model (CC vs. CT + TT), the effect was also observed (OR = 2.70; 95% CI: 1.28–5.69; *p* = 0.011). In the log-additive model, a linear relationship was identified between the number of C alleles and bleeding risk (OR = 1.93; 95% CI: 1.17–3.20; *p* = 0.009). After multiple-testing correction, the log-additive model remained significant with both FDR (q = 0.0275) and Bonferroni adjustment (p_adj = 0.044), while the codominant (q = 0.045) and recessive (q = 0.0275) models retained significance only under FDR but not Bonferroni correction.

For the other *ABCB1* polymorphisms (rs2032582 and rs1128503), no statistically significant association with bleeding frequency was identified. Although differences in complication rates were observed between genotypes, they did not reach statistical significance, which may be attributable to the limited statistical power of the sample as well as the multifactorial influences on apixaban exposure.

In addition to the analysis of individual SNPs, a haplotype analysis of three *ABCB1* polymorphisms (rs2032582, rs1045642, rs1128503) was performed (Table 6).

The most frequent haplotypes in the studied cohort were G–C–C (40.5%), T–T–T (36.5%), and G–T–C (13.0%). The global haplotype association test did not reach statistical significance (*p* = 0.061). Although nominal *p*-values suggested a protective trend for the T–T–T (OR = 0.61; 95% CI: 0.35–1.07; *p* = 0.089) and G–T–C haplotypes (OR = 0.42; 95% CI: 0.16–1.09; *p* = 0.077), these associations did not achieve statistical significance.

Polymorphisms in cytochrome P450 enzymes also did not demonstrate a significant association with bleeding risk. Specifically, for the CYP3A5*3 (rs776746) variant, only a trend was observed: heterozygous carriers (GA) had a higher frequency of bleeding compared with GG homozygotes (32.1% vs. 18.2%); however, the difference did not reach statistical significance (OR = 2.15; 95% CI: 0.89–5.21; *p* = 0.086). This may indicate a potential role of CYP3A5 in the interindividual variability of apixaban metabolism, which requires confirmation in larger cohorts. For the rare CYP3A4*22 (rs35599367) variant, no association with hemorrhagic complications was identified, most likely due to the very low prevalence of this polymorphism in the European population.

### 3.3. Assessment of the Contribution of Polypharmacy to Bleeding Frequency

Analysis of concomitant therapy (Table A1 in Appendix A) showed that most classes of medications included in patient treatment regimens were not significantly associated with bleeding risk. The use of ACE inhibitors, angiotensin II receptor blockers, various groups of diuretics (thiazide, loop, potassium-sparing), hypoglycemic agents, and proton pump inhibitors was not linked to a significant increase in the likelihood of hemorrhagic complications (*p* > 0.05).

The use of antiplatelet agents was not associated with an elevated bleeding risk (OR = 1.83; 95% CI: 0.53–6.27; *p* = 0.332). Similarly, calcium channel blockers (OR = 1.49; 95% CI: 0.73–3.03; *p* = 0.267) and angiotensin II receptor blockers (OR = 1.86; 95% CI: 0.83–4.18; *p* = 0.131) did not demonstrate a statistically significant association with bleeding events.

In contrast, a possible association was observed for antiarrhythmic drugs, where their use appeared to be linked to a more than a threefold increase in bleeding risk (OR = 3.12; 95% CI: 1.11–8.80; *p* = 0.025). Statin therapy also suggested an increased risk of hemorrhagic complications (OR = 2.84; 95% CI: 1.18–6.82; *p* = 0.016).

When stratified by individual molecules within the antiarrhythmic drug group (amiodarone, *n* = 5; digoxin, *n* = 2) and statins (simvastatin, *n* = 8; rosuvastatin, *n* = 28), the very small sample sizes preclude firm conclusions. A nominally significant association with bleeding risk persisted only for rosuvastatin (OR = 2.189; 95% CI: 1.039–4.612; *p* = 0.037), but this finding should be interpreted with caution and regarded as exploratory.

### 3.4. Assessment of Pharmacokinetic Parameters

**Figure 1:**
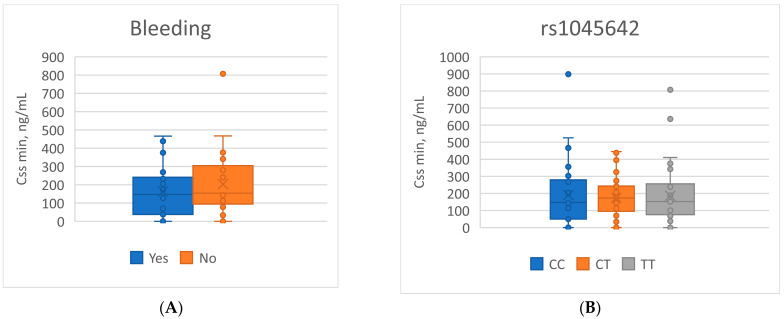
Boxplots of Cssmin apixaban concentrations related to: (**A**) the presence of bleeding events; (**B**) the rs1045642 genotype.

Analysis of the minimum steady-state concentration of apixaban (Cssmin) showed that mean plasma exposure levels did not differ significantly depending on the occurrence of bleeding events. In patients with complications, the mean Cssmin was 164.01 ± 136.26 ng/mL, whereas in the group without bleeding it was 185.67 ± 154.26 ng/mL (*p* = 0.505). Stratification by genotypes of the *ABCB1* polymorphisms (rs2032582, rs1045642, rs1128503), CYP3A5*3 (rs776746), and CYP3A4*22 (rs35599367) also revealed no significant differences in Cssmin (*p* > 0.05 for all comparisons). Among carriers of risk alleles previously associated with increased bleeding frequency (the CC variant of *ABCB1* rs1045642), mean apixaban plasma concentrations did not differ from those observed in other genotypes (Figure 1, Table 7).

## 4. Discussion

Our study was devoted to assessing the impact of polymorphic variants in genes encoding the P-glycoprotein transporter and cytochrome P450 enzymes on apixaban exposure (Cssmin) and the risk of hemorrhagic complications in elderly patients with NVAF receiving therapy under conditions of polypharmacy. In the initial analysis, we observed an association between the *ABCB1* rs1045642 (3435C>T) polymorphism and bleeding risk: carriers of the homozygous CC genotype showed a nearly threefold higher likelihood of bleeding events compared with carriers of the alternative allelic variants. However, after correction for multiple testing, only the log-additive model of rs1045642 remained significant, whereas other inheritance models and polymorphisms (*ABCB1* rs2032582, rs1128503; CYP3A5*3 rs776746; CYP3A4*22 rs35599367) did not retain statistical significance. At the same time, none of the studied genetic markers, including rs1045642, showed a significant effect on the Cssmin of apixaban.

Apixaban is mainly metabolized by CYP3A4/3A5 and transported by P-glycoprotein (P-gp), with intra- and interindividual variability in exposure are estimated at approximately 20% and 30%, respectively [8,22]. Clinical factors contribute to variability but do not fully explain interindividual differences, highlighting the role of genetic determinants. Among these, common *ABCB1* variants (3435T>C, 2677T>G/A, 1236T>C) form haplotypes associated with altered P-gp function, typically resulting in reduced activity and substrate specificity [23] and have been widely investigated in pharmacogenetic studies of DOACs.

The evidence regarding the clinical impact of these variants remains inconsistent. In the study by Kryukov A.V. et al. [24], no significant effect of *ABCB1* rs1045642 or rs4148738, nor of CYP3A5*3 rs776746, was observed on apixaban pharmacokinetics in patients receiving 10 mg/day. Similarly, in a cohort of 44 Japanese patients, Ueshima S. et al. [10] reported that none of the *ABCB1* polymorphisms (1236C>T, 2677G>T/A, 3435C>T) affected the concentration/dose ratio (C/D) of apixaban in plasma. In line with these findings, Skripka A. et al. (2024) in a cohort of 84 AF patients treated with apixaban also found no significant associations between *ABCB1* rs1045642, rs4148738, CYP3A4*22, or CYP3A5*3 variants and either bleeding events or C/D values [17].

In contrast, a meta-analysis by Xie Q. et al. [14], which included 535 DOAC users, demonstrated that carriers of the rs1045642 CC genotype had lower maximum plasma concentrations (Cmax) compared with TT carriers, as well as lower AUC_0–∞_ compared with T allele carriers. In the same analysis, rs2032582 GG carriers exhibited lower Cmax values than A/T allele carriers [14]. Comparable results were obtained in a more recent meta-analysis by Shi J. et al. (2023) [15]. Conversely, in a cohort of 164 AF patients receiving apixaban, Lenoir C. et al. (2022) found that none of the *ABCB1* variants (1236C>T, 2677G>T/A, 3435C>T) influenced apixaban AUC [12]. On the other hand, data from two smaller studies suggested that the intronic *ABCB1* rs4148738 variant was associated with lower peak apixaban concentrations [25] and a reduced risk of bleeding in apixaban-treated patients [18]. For rs1045642, patients with the AA genotype had a higher bleeding risk compared with G allele carriers (38.7% vs. 19.9%, *p* = 0.017), as shown in a cohort of AF patients receiving apixaban and rivaroxaban [16]. However, a larger GWAS did not confirm a consistent correlation between *ABCB1* variants and hemorrhagic events [13].

The results of our study suggest that the association between bleeding risk and the CC genotype of rs1045642 may indeed exist, highlighting the potential clinical relevance of this marker, particularly in patients with multiple risk factors. Our analysis of different inheritance models for rs1045642 provides further insight into the nature of its association with bleeding events. The strongest signal was observed in the log-additive model (OR = 1.93 per C allele; 95% CI: 1.17–3.20; *p* = 0.0088), which remained statistically significant after both FDR and Bonferroni correction. The recessive (OR = 2.70 for CC versus TT + CT; 95% CI: 1.28–5.69; *p* = 0.011) and codominant (3.50 for CC vs. TT and TC; 95% CI:1.30–9.45; *p* = 0.027) models also suggested increased risk, but these associations retained significance only under FDR correction and not after Bonferroni adjustment. This pattern supports an additive inheritance model, whereby each additional C allele increases bleeding risk, while indicating that more stringent corrections attenuate the evidence for the recessive effect. In contrast, heterozygous carriers (CT genotype) did not exhibit a significantly different bleeding risk compared with TT homozygotes.

Notably, consistent with the studies by Kryukov A.V. [24], Ueshima S. [10], and Skripka A. [17], we did not observe a significant effect of rs1045642 on the Cssmin of apixaban. This apparent discrepancy between the clinical outcome (bleeding events) and the pharmacokinetic parameter (Cssmin) suggests that the mechanism by which this marker exerts its influence may not be mediated solely through changes in systemic drug exposure. Although the global haplotype association test did not reach strict statistical significance (*p* = 0.061), the observed trends for the T–T–T haplotype (OR = 0.61; 95% CI: 0.35–1.07; *p* = 0.089) and G–T–C (OR = 0.42, 95% CI: 0.16–1.09; *p* = 0.077) toward lower bleeding risk are consistent with the literature indicating that haplotypes, rather than individual SNPs, serve as better predictors of P-glycoprotein function [26,27]. 

The observed association between *ABCB1* rs1045642 (CC) and bleeding was not accompanied by differences in steady-state Cssmin, suggesting that a systemic trough-exposure mechanism is unlikely to fully explain the signal. Several non-mutually exclusive hypotheses warrant consideration. First, our pharmacokinetic assessment captured only trough concentrations; however, bleeding liability with factor-Xa inhibitors may relate more closely to peak exposure (Cmax) or overall exposure (AUC), neither of which was measured here, potentially obscuring exposure-response relationships detectable at peaks rather than troughs [8,22]. Second, rs1045642 (3435C>T) tags haplotypes linked to P-gp function; *ABCB1* primarily governs barrier/tissue transport (e.g., intestinal epithelium, renal tubules, endothelium) rather than plasma disposition per se, so genotype-dependent tissue distribution (including at bleeding-prone mucosal sites) could increase local anticoagulant effect without altering Cssmin [23,28]. Third, under conditions of polypharmacy, *ABCB1* variation may modulate the impact of drug–drug interactions with P-gp/CYP3A inhibitors (e.g., amiodarone, certain statins), amplifying bleeding risk independently of trough levels—an interpretation consistent with our DDI findings and with the recognized interaction profile of apixaban [8,22]. Finally, in very elderly patients, the multifactorial nature of bleeding risk (renal function, platelet count, frailty/comorbidity) can interact with genetic predisposition, yielding a phenotype not mediated by Cssmin alone. Collectively, these considerations argue against over-interpreting a genotype → Cssmin → bleeding pathway and support a model in which tissue-level transport, peak/overall exposure, and DDIs jointly shape risk; future multicenter studies incorporating Cmax/AUC, standardized bleeding definitions, and multivariable adjustment are needed to test these hypotheses prospectively.

CYP3A4 is the primary enzyme responsible for apixaban metabolism. CYP3A4 activity varies considerably among individuals due to genetic differences, environmental factors, health status, and comorbid conditions. The CYP3A4*22 (rs35599367 C>T) variant, located in intron 6 of the CYP3A4 gene, leads to alternative splicing and reduces CYP3A4 expression and activity by 40–50% [29], which can contribute to substantial interindividual variability in substrate metabolism. CYP3A4*22 occurs more frequently in European populations, with a minor allele frequency of approximately 5%, whereas its prevalence is <0.6% in Asian populations [30]. Carriers of the CYP3A4*22 variant may exhibit reduced CYP3A4 metabolic activity and, consequently, increased substrate exposure. Despite the central role of CYP3A4 in apixaban metabolism, no evidence has been found that the CYP3A4*22 variant significantly influences apixaban plasma concentrations or clinical outcomes [12,13], which is consistent with our findings. On the other hand, it should be noted that this intronic variant explains only up to 12% of CYP3A4 variability [31], and its effect may be offset given the multifactorial nature of variability in pharmacological response. In our cohort, the low-function CYP3A4*22 variant was represented exclusively by heterozygous carriers (CT genotype, 5.46%). No T homozygotes were detected, which is also characteristic of European populations. The low frequency of this variant did not allow for definitive conclusions regarding its influence on apixaban exposure or safety profile.

The CYP3A5*3 (rs776746) variant encodes a loss-of-function enzyme: heterozygous carriers (AG) have moderately reduced metabolism, while GG homozygotes do not express CYP3A5. This may represent a risk factor for adverse reactions, particularly bleeding, during apixaban therapy [32]. In our study, we did not detect a statistically significant effect of the CYP3A5*3 rs776746 polymorphism on either Cssmin or the risk of hemorrhagic complications. On the one hand, studies by Ueshima S. et al. (2017, 2018) demonstrated a positive association of ABCG2 rs2231142 and CYP3A5*3 rs776746, in combination with renal function, with the concentration-to-dose ratio of apixaban in Japanese patients with atrial fibrillation [10,11]. Such differences are likely attributable both to the ethnic specificity of functional allele distribution and to sample size limitations. Our findings are consistent with those of Lenoir C. et al. (2022) [12] and Attelind S. et al. (2022) [13], who also found no significant contribution of CYP3A5*3 rs776746 to apixaban exposure in patients predominantly of European ancestry. On the other hand, apixaban is primarily a substrate of CYP3A4. In the absence of functional CYP3A5 protein, its role in apixaban metabolism may be compensated by increased activity of the CYP3A4 isoenzyme, minimizing pharmacokinetic differences between carriers of different CYP3A5*3 genotypes. Moreover, since all patients received multiple concomitant medications, the strong influence of CYP3A4/P-gp inhibitors or inducers could have masked more subtle genetic effects of CYP3A5*3.

Analysis of concomitant therapy showed that most drug classes (ACE inhibitors, angiotensin II receptor blockers, diuretics, hypoglycemic agents, proton pump inhibitors) did not significantly influence the frequency of hemorrhagic complications. However, the use of certain pharmacological groups emerged as important modifying risk factors. In particular, the use of antiarrhythmic drugs was associated with more than a threefold increase in the incidence of bleeding events (OR = 3.12; 95% CI: 1.11–8.80; *p* = 0.025). It should be noted that this association was based on a relatively small subgroup of patients (amiodarone, *n* = 5; digoxin, *n* = 2). Given the well-established ability of amiodarone to inhibit CYP3A4 and P-glycoprotein, its contribution appears to be key, as it increases the exposure of substrates, including apixaban. By contrast, digoxin—being a P-gp substrate—is likely to exert a less pronounced effect on apixaban exposure. Another significant factor was statin therapy (OR = 2.84; 95% CI: 1.18–6.82; *p* = 0.016). When stratified by individual molecules, a statistically significant association persisted only for rosuvastatin (*n* = 28; OR = 2.19; 95% CI: 1.04–4.61; *p* = 0.037), whereas simvastatin (*n* = 8) did not show an increase in incidence of bleeding events. The observed associations with amiodarone and rosuvastatin likely reflect drug–drug interactions via CYP3A4 and P-gp, consistent with known mechanisms. These findings emphasize that specific co-medications, rather than polypharmacy per se, shape bleeding risk.

Limitations. Our study has several limitations that should be considered when interpreting the findings. First, this was a cross-sectional observational study, and, therefore, it carries the inherent limitations of this design. Second, the single-center prospective design and inclusion of hospitalized patients limit external validity and may introduce selection bias; therefore, the conclusions require validation in multicenter cohorts with more diverse profiles of comorbidity and medication use. Third, although the overall sample size was comparable to other pharmacogenetic studies (*n* = 197), the number of clinical events was moderate (40 bleeding cases), and the frequencies of CYP3A4/5 genetic variants were low. This reduces statistical power to detect small-to-moderate effects and increases the risk of type II error in subgroup analyses. Fourth, the subgroup analyses of potential drug–drug interactions (e.g., amiodarone *n* = 5, digoxin *n* = 2, rosuvastatin *n* = 28, simvastatin *n* = 8) were based on very small sample sizes, which substantially limits the precision of effect estimates; therefore, these findings should be regarded as exploratory and interpreted with caution. Moreover, given the limited sample size and broad spectrum of concomitant medications per patient, it was not feasible to adequately evaluate the combined effects of drug–drug interactions and genetic polymorphisms on apixaban-related complications. Fifth, the pharmacokinetic assessment was limited to measuring Cssmin without measuring the Cmax and AUC; in addition, residual variability in blood sampling time relative to the last dose cannot be excluded. These factors may have reduced the sensitivity of the analysis to subtle genetic and pharmacological influences on apixaban exposure. Due to the relatively small sample size and limited number of bleeding events, multivariable logistic regression adjusting for clinical covariates could not be reliably performed. Data on dietary supplements and lifestyle factors were not systematically collected, which may represent an unmeasured source of variability.

Our findings should be interpreted as confirmatory rather than mechanistic. The study contributes a single-center replication dataset from an underrepresented population—elderly NVAF patients under conditions of polypharmacy—thereby extending the heterogeneous evidence base on DOAC pharmacogenetics. While we identified an association of *ABCB1* rs1045642 with bleeding risk, this effect was not mediated through systemic apixaban exposure (Cssmin). Thus, the results should be viewed as hypothesis-generating and supportive of the need for larger multicenter and multi-ethnic studies incorporating comprehensive pharmacokinetic assessment (Cmax, AUC), standardized bleeding definitions, and multivariable analyses.

## 5. Conclusions

Our study demonstrated that, in elderly patients, variability in the clinical response to apixaban is determined by a combination of genetic and pharmacological factors. Specifically, our results provide new knowledge by showing that the *ABCB1* rs1045642 CC genotype might be associated with an increased bleeding risk in very elderly patients under polypharmacy, independently of trough apixaban concentrations, thereby suggesting mechanisms beyond systemic exposure. In contrast, no significant influence was identified for CYP3A4 and CYP3A5 variants. Concomitant pharmacotherapy—particularly the use of agents with known pharmacokinetic interactions—played a substantial role in shaping the risk of complications. While not offering new mechanistic insight, this work extends previous heterogeneous findings to a unique elderly, polypharmacy Russian cohort. These results highlight the need for larger multicenter studies to validate and refine personalized anticoagulant strategies. We believe, the integration of genetic data with clinical and pharmacokinetic characteristics may form the basis for personalized algorithms of DOAC prescription, aimed at optimizing the benefit–risk balance in elderly patients and in the context of polypharmacy.

## Figures and Tables

**Table 1:** Clinical and demographic characteristics of the study cohort and comparison by outcome.

Parameter	Cohort (*n* = 197)	Comparison by Outcome		*p*-Value	Women (*n* = 132)		*p*-Value	Men (*n* = 65)		*p*-Value
		No Bleeding (*n* = 157)	Bleeding (*n* = 40)		No Bleeding (*n* = 106)	Bleeding (*n* = 26)		No Bleeding (*n* = 51)	Bleeding (*n* = 14)	
Age, years	83.17 ± 8.58	83.32 ± 8.90	82.63 ± 7.28	0.65	83.72 ± 8.71	82.00 ± 6.27	0.346	82.49 ± 9.29	83.79 ± 8.99	0.643
Height, m	1.64 ± 0.09	1.64 ± 0.09	1.65 ± 0.09	0.299	1.59 ± 0.06	1.60 ± 0.05	0.214	1.73 ± 0.06	1.74 ± 0.07	0.721
Weight, kg	75.45 ± 16.93	75.78 ± 17.18	74.18 ± 16.05	0.594	72.50 ± 16.45	69.15 ± 14.57	0.344	82.59 ± 16.81	83.50 ± 14.86	0.854
BMI, kg/m^2^	28.04 ± 5.63	28.30 ± 5.83	27.02 ± 4.70	0.200	28.71 ± 6.16	26.80 ± 5.17	0.145	27.43 ± 5.01	27.43 ± 3.82	0.999
GFR, mL/min/1.73 m^2^	49.30 ± 21.15	49.92 ± 21.73	46.88 ± 18.80	0.418	46.14 ± 18.66	45.50 ± 18.49	0.875	57.76 ± 25.46	49.43 ± 19.79	0.261
Hemoglobin, g/L	118.29 ± 19.24	118.86 ± 18.98	116.05 ± 20.36	0.411	117.81 ± 17.82	116.38 ± 18.79	0.717	121.04 ± 21.20	115.43 ± 23.73	0.395
Leukocytes, ×10^9^/L	6.66 ± 2.38	6.60 ± 2.41	6.93 ± 2.25	0.427	6.62 ± 2.57	7.06 ± 2.44	0.430	6.54 ± 2.06	6.69 ± 1.90	0.813
Erythrocytes, ×10^12^/L	4.13 ± 0.64	4.14 ± 0.66	4.09 ± 0.58	0.657	4.18 ± 0.63	4.08 ± 0.61	0.462	4.07 ± 0.71	4.12 ± 0.56	0.804
Platelets, ×10^9^/L	223.97 ± 75.36	218.29 ± 69.53	246.30 ± 92.55	**0.035** *	227.87 ± 72.99	238.54 ± 50.80	0.482	198.39 ± 57.41	260.71 ± 142.78	**0.015** *
Urea, mmol/L	9.57 ± 9.39	9.70 ± 10.24	9.06 ± 4.78	0.698	8.40 ± 3.70	9.15 ± 5.22	0.397	12.41 ± 16.95	8.89 ± 4.01	0.445
Creatinine, μmol/L	106.45 ± 34.38	105.13 ± 32.24	111.66 ± 41.81	0.284	102.29 ± 29.83	102.90 ± 39.88	0.931	111.03 ± 36.34	127.92 ± 41.79	0.140

Note: *—Statistically significant *p*-values were observed; Baseline clinical and laboratory comparisons were considered descriptive and not corrected for multiple testing.

**Table 2:** Association between CKD stage and the risk of bleeding events.

CKD Stage	Total (*n*)	Bleeding Events, *n* (%)	No Bleeding, *n* (%)	χ^2^	*p*-Value	OR (95% CI)
Stage 2	58	10 (17.2%)	48 (82.8%)	0.477	0.490	0.757 (0.343–1.671)
Stage 3a	66	16 (24.2%)	50 (75.8%)	0.951	0.330	1.427 (0.697–2.920)
Stage 3b	19	4 (21.1%)	15 (78.9%)	0.007	0.933	1.052 (0.329–3.362)
Stage 4	54	10 (18.5%)	44 (81.5%)	0.147	0.702	0.856 (0.386–1.897)

**Table 3:** Distribution of genotypes of the studied markers in the cohort and conformity to the Hardy–Weinberg equilibrium.

Polymorphism	Total (*n*)	Genotype	Frequency			MAF, %	χ^2^	*p*-Value
			Observed (*n*)	Expected (*n*)	%			
*ABCB1* (rs2032582, 2677G>T/A)	195	GG	70	64.33	35.90	42.6	2.760	0.252
		GT	84	95.34	43.08			
		TT	41	35.33	21.03			
*ABCB1* (rs1045642, 3435C>T)	194	CC	47	44.10	24.23	52.3	0.695	0.707
		CT	91	96.79	46.91			
		TT	56	53.10	28.87			
*ABCB1* (rs1128503, 1236C>T)	195	CC	66	65.48	33.85	42.1	0.023	0.988
		CT	94	95.04	48.21			
		TT	35	34.48	17.95			
CYP3A5*3 (rs776746, 6986A>G)	194	AA	1	1.16	0.52	92.3	0.026	0.987
		GA	28	27.68	14.43			
		GG	165	165.16	85.05			
CYP3A4*22 (rs35599367, C>T)	183	CC	173	173.14	94.54	2.7	0.144	0.930
		CT	10	9.73	5.46			
		TT	0	0.14	0.00			

**Table 4:** Association of *ABCB1*, *CYP3A4*, and *CYP3A5* polymorphisms with the risk of bleeding events.

Polymorphism	Genotype	Bleeding Events, *n* (%)	No Bleeding, *n* (%)	χ2	*p*-Value	OR (95% CI)
*ABCB1* (rs2032582, 2677G>T/A)	GG	17 (43.6)	53 (34)	1.254	0.263	1.502 (0.735–3.068)
	GT	15 (38.5)	69 (44.2)	0.424	0.515	0.788 (0.384–1.616)
	TT	7 (17.9)	34 (21.8)	0.278	0.598	0.785 (0.319–1.934)
*ABCB1* (rs1045642, 3435C>T)	CC	16 (41)	31 (19.9)	7.632	**0.006** *	2.805 (1.326–5.935)
	CT	16 (41)	75 (48.4)	0.678	0.410	0.742 (0.364–1.512)
	TT	7 (17.9)	49 (31.4)	2.762	0.097	0.478 (0.197–1.157)
*ABCB1* (rs1128503, 1236C>T)	CC	15 (38.5)	51 (32.7)	0.464	0.499	1.287 (0.622–2.661)
	CT	19 (48.7)	75 (48.1)	0.005	0.943	1.026 (0.508–2.070)
	TT	5 (12.8)	30 (19.2)	0.871	0.351	0.618 (0.223–1.712)
CYP3A5*3 (rs776746, 6986A>G)	GG	30 (76.9)	135 (87.1)	2.537	0.111	0.494 (0.205–1.191)
	GA	9 (22.5)	19 (12.1)	2.953	0.086	2.147 (0.885–5.209)
	AA	0 (0)	1 (0.6)	NA	>0.05	NA
CYP3A4*22 (rs35599367, C>T)	CC	36 (20.8)	137 (79.2)	0.685	0.408	0.423 (0.052–3.447
	CT	1 (10.0)	9 (90)	NaN	>0.05 ^$^	2.365 (0.290–19.281)

Note: NA—not calculated due to the absence of events in one of the groups; $ *p*-value corresponds to Fisher’s exact test; NaN—for Fisher’s exact test, the test statistic is not computed, and only the *p*-value is reported; *—Statistically significant *p*-values were observed, but no associations remained significant after correction for multiple testing (FDR or Bonferroni).

**Table 5:** Association analysis of rs1045642 with bleeding events under different inheritance models.

Model	Genotype	Bleeding (%)	No Bleeding (%)	OR (95% CI)	*p*-Value	FDR q-Value	Bonferroni Adjustment
Codominant	T/T	7 (17.9%)	49 (31.4%)	1.00	**0.027** *	**0.045** *	0.135
	C/T	16 (41%)	75 (48.1%)	1.49 (0.57–3.89)			
	C/C	16 (41%)	32 (20.5%)	3.50 (1.30–9.45)			
Dominant	T/T	7 (17.9%)	49 (31.4%)	1.00	0.085	0.106	0.425
	C/T-C/C	32 (82%)	107 (68.6%)	2.09 (0.86–5.07)			
Recessive	T/T-C/T	23 (59%)	124 (79.5%)	1.00	**0.011** *	**0.0275** *	0.055
	C/C	16 (41%)	32 (20.5%)	2.70 (1.28–5.69)			
Overdominant	T/T-C/C	23 (59%)	81 (51.9%)	1.00	0.43	0.430	1.000
	C/T	16 (41%)	75 (48.1%)	0.75 (0.37–1.53)			
Log-additive	---	---	---	1.93 (1.17–3.20)	**0.0088** *	**0.0275** *	**0.044**

Note: *—Statistically significant *p*-values were observed; q-values calculated by the Benjamini–Hochberg false discovery rate (FDR); Bonferroni adjustment based on five inheritance models.

**Table 6:** Haplotype analysis of three *ABCB1* polymorphisms (rs2032582, rs1045642, rs1128503) and their association with bleeding frequency.

#	rs2032582	rs1045642	rs1128503	Overall Frequency	Bleeding Frequency	No Bleeding Frequency	OR (95% CI)	*p*-Value
1	G	C	C	0.405	0.534	0.376	1.00	---
2	T	T	T	0.365	0.305	0.381	0.61 (0.35–1.07)	0.089
3	G	T	C	0.131	0.068	0.143	0.42 (0.16–1.09)	0.077
4	G	C	T	0.038	0.015	0.042	0.48 (0.10–2.33)	0.36
5	T	T	C	0.024	0	0.030	0.00 (−Inf–Inf)	1
6	T	C	C	0.019	0.026	0.018	1.51 (0.27–8.55)	0.64
7	T	C	T	0.017	0.040	0.010	2.75 (0.42–18.10)	0.29
Global haplotype association *p*-value								0.061

**Table 7:** Comparative analysis of apixaban Cssmin values.

Factor	Category		Yes	No	*p*-Value
Bleeding events	Yes	*n*	27	110	0.505
	No	mean ± SD	164.01 ± 136.26	185.67 ± 154.26	
*ABCB1* (rs2032582, 2677G>T/A)	GG	*n*	51	86	0.884
		mean ± SD	183.84 ± 170.97	179.95 ± 138.25	
	GT	*n*	59	78	0.763
		mean ± SD	185.89 ± 146.97	178.00 ± 154.23	
	TT	*n*	27	110	0.581
		mean ± SD	166.98 ± 118.49	184.94 ± 157.81	
*ABCB1* (rs1045642, 3435C>T)	CC	*n*	31	106	0.699
		mean ± SD	190.63 ± 190.66	178.70 ± 137.76	
	CT	*n*	64	73	0.599
		mean ± SD	174.14 ± 114.47	187.77 ± 176.96	
	TT	*n*	42	95	0.826
		mean ± SD	185.65 ± 168.48	179.52 ± 142.98	
*ABCB1* (rs1128503, 1236C>T)	CC	*n*	50	87	0.515
		mean ± SD	192.49 ± 172.91	175.03 ± 136.91	
	CT	*n*	63	74	0.737
		mean ± SD	176.69 ± 144.46	185.40 ± 156.60	
	TT	*n*	24	113	0.701
		mean ± SD	170.65 ± 117.50	183.68 ± 157.14	
CYP3A5*3 (rs776746, A7986G)	GG	*n*	117	20	0.816
		mean ± SD	182.64 ± 158.92	174.15 ± 90.56	
	GA	*n*	19	118	0.900
		mean ± SD	177.36 ± 91.86	182.05 ± 158.37	
	AA	*n*	1	136	0.651
		mean ± SD	113.16 ± 0.01	181.90 ± 151.09	
CYP3A4*22 (rs35599367, C>T)	CC	*n*	124	7	0.786
		mean ± SD	182.14 ± 153.12	198.24 ± 139.88	
	CT	*n*	7	124	0.786
		mean ± SD	198.24 ± 139.88	182.14 ± 153.12	

## Data Availability

The datasets generated and analyzed during this study are not publicly available due to ethical restrictions and patient confidentiality protections under Russian Federation laws on personal data protection (Federal Law No. 152-FZ). However, anonymized data supporting the findings may be made available upon reasonable request from qualified researchers, subject to approval by the Local Ethics Committee of the War Veterans Hospital No. 2 of the Moscow Healthcare Department (contact: gvv2@zdrav.mos.ru). Requests should include a detailed research proposal and data protection plan.

## Abbreviations

The following abbreviations are used in this manuscript:

AF	Atrial fibrillation
NVAF	Non-valvular atrial fibrillation
DOACs	Direct oral anticoagulants
INR	International normalized ratio
CKD	Chronic kidney disease
GFR	Glomerular filtration rate
OR	Odds ratio
CI	Confidence interval
Cssmin	Minimum steady-state plasma concentration
C/D	Concentration-to-dose ratio
Cmax	Maximum plasma concentration
AUC	Area under the curve
ADME	Absorption, distribution, metabolism, and excretion
P-gp	P-glycoprotein
PCR	Polymerase chain reaction
HPLC	High-performance liquid chromatography
MS/MS	Tandem mass spectrometry
GWAS	Genome-wide association study

## Appendix A

**Table A1:** Association of concomitant medication use with bleeding frequency in patients receiving apixaban.

Medication Class		Bleeding Event		χ^2^	*p*-Value	OR (95% CI)
		Yes (%)	No (%)			
Beta-blockers	Yes	40 (100)	157 (100)	NA	NA	NA
	No	0 (0)	0 (0)			
ACE inhibitors	Yes	8 (20)	37 (23.6)	0.230	0.631	0.811 (0.344–1.912)
	No	32 (80)	120 (76.4)			
Proton pump inhibitors	Yes	13 (32.5)	40 (25.5)	0.799	0.371	1.408 (0.663–2.990)
	No	27 (67.5)	117 (74.5)			
Thiazide diuretics	Yes	4 (10)	19 (12.1)	0.137	0.712	0.807 (0.258–2.521)
	No	36 (90)	138 (87.9)			
Loop diuretics	Yes	26 (65)	105 (66.9)	0.051	0.822	0.920 (0.443–1.908)
	No	14 (35)	52 (33.1)			
Potassium-sparing diuretics	Yes	19 (47.5)	74 (47.1)	0.002	0.967	1.015 (0.506–2.034)
	No	21 (52.5)	83 (52.9)			
Carbonic anhydrase inhibitors	Yes	1 (2.5)	5 (3.2)	0.051	0.822	0.779 (0.088–6.866)
	No	39 (97.5)	152 (96.8)			
Hypoglycemic agents	Yes	12 (30)	35 (22.3)	1.042	0.307	1.494 (0.689–3.238)
	No	28 (70)	122 (77.7)			
Antiplatelet agents	Yes	4 (10)	9 (5.7)	0.942	0.332	1.827 (0.533–6.269)
	No	36 (90)	148 (94.3)			
Direct anticoagulants	Yes	1 (2.5)	5 (3.2)	0.051	0.822	0.779 (0.088–6.866
	No	39 (97.5)	152 (96.8)			
Antiarrhythmic drugs	Yes	7 (17.5)	10 (6.4)	5.009	**0.025** *	3.118 (1.105–8.796)
	No	33 (82.5)	147 (93.6)			
Calcium channel blockers	Yes	17 (42.5)	52 (33.1)	1.232	0.267	1.492 (0.734–3.034)
	No	23 (57.5)	105 (66.9)			
Angiotensin II receptor blockers	Yes	31 (77.5)	102 (65)	2.283	0.131	1.857 (0.825–4.181)
	No	9 (22.5)	55 (35)			
HMG-CoA reductase inhibitors (statins)	Yes	33 (82.5)	98 (62.4)	5.769	**0.016** *	2.838 (1.181–6.824)
	No	7 (17.5)	59 (37.6)			
NSAIDs	Yes	6 (15)	45 (28.7)	3.101	0.078	0.439 (0.173–1.118)
	No	34 (85)	112 (71.3)			

Note: * statistically significant *p*-values were observed.
